# Structural failure in bridging stentgrafts for branched endovascular aneurysm repair: a case–control study

**DOI:** 10.1186/s13244-022-01196-6

**Published:** 2022-03-28

**Authors:** Sven R. Hauck, Alexander Kupferthaler, Martin C. Freund, Peter Pichler, Marie-Elisabeth Stelzmüller, Christopher Burghuber, Marek Ehrlich, Harald Teufelsbauer, Christian Loewe, Martin A. Funovics

**Affiliations:** 1 Division of Cardiovascular and Interventional Radiology, Department of Bioimaging and Image-Guided Therapy, Medical University of Vienna, Vienna, Austria; 2 Department of Diagnostic and Interventional Radiology, Ordensklinikum Linz & Medical Faculty, Johannes Kepler University, Linz, Austria; 3 Department of Radiology, Medical University Innsbruck and Tirol Kliniken GmbH, Innsbruck, Austria; 4 Department of Radiology, Johannes Kepler University, Linz, Austria; 5 Department of Cardiothoracic Surgery, Medical University of Vienna, Vienna, Austria; 6 Division of Vascular Surgery, Department of Surgery, Medical University of Vienna, Vienna, Austria

**Keywords:** Bridging stentgraft, Structural failure, Branched EVAR, Type 3 endoleak, bEVAR complication

## Abstract

**Objective:**

To present a case series of spontaneous structural failure of bridging stentgrafts (BSGs) after branched endovascular aortic repair (bEVAR), as well as their failure types and their detection. While bEVAR is a safe and effective procedure, one main limitation is the reintervention rate associated with the BSGs. Structural failure of BSGs, defined as fabric disruption, stent fracture with leak or complete separation is a major cause for reinterventions and difficult to detect in computed tomography angiography (CTA).

**Methods:**

From a multicenter bEVAR complication database, structural BSG failures were identified. Patient and BSG characteristics, detection mode, failure type, treatment and outcome were recorded and compared with bEVAR patients with intact BSGs.

**Results:**

Twenty-three BSG failures were detected in 12 patients with only 43% directly identified in CTA, after a mean of 21.4 months after implantation. The BSGs were Advanta (*n* = 4), E-Ventus (*n* = 16) and BeGraft (*n* = 3) in 10 renal, 9 superior mesenteric, and 4 celiac branches. Religning with another BSG was successful in 20/22 cases, one BSG failure necessitated renal branch embolization (organ loss), and one mesenteric bypass surgery. Two reintervention-related mortalities occurred.

**Conclusion:**

Structural failure of BSGs is a serious limitation for bEVAR, which can result in high reintervention rates and serious complications. BSG failure typically occurs in single-layer types and events are clustered in patients. The necessary reinterventions carry serious morbidity and mortality. Since the use as BSG in bEVAR is off-label with all current BSG manufacturers, caution is advised regarding patient-informed consent.

## Key points

- 23 cases of bridging stentgraft failures after branched EVAR were identified.
- In 57% the failures were not identified as such in the CT scans.
- 22% were incidental findings at angiography.
- Repair was associated with mortality and serious complications.
- Avoiding single-layer bridging stentgrafts and more frequent CT surveillance after one bridging stentgraft failure may improve outcome.

## Introduction

The introduction of fenestrated (fEVAR) and branched (bEVAR) endovascular aortic repair (EVAR) over the last two decades has expanded the applicability of endovascular aneurysm repair to practically all regions of the aorta [1–3]. With the refinement of the materials used and increasing expertise of operators, technical success rates, complication rates and long-term stability of aneurysm exclusion approach those of conventional EVAR [4–7].

However, bEVAR is not free from technological weaknesses [8, 9]: One of the reasons that lead to higher reintervention rates in bEVAR are the bridging stentgrafts (BSGs), which are used to connect the branches or fenestrations of the aortic main body with the ostia of the visceral arteries [10, 11]. BSGs are associated with a reintervention rate of up to 33% after 2–3 years and up to 50% after 5 years [12–14]. While there are several manufacturers producing stentgrafts of suitable sizes, none of them labelled the application as BSGs on the product instructions for use. Devices have entered clinical use before being specifically tested for their durability as BSGs and all products are currently used off-label for this special purpose [14, 15].

Between 2014 and 2019, we observed an unusually high number of BSG failures (BSG leaks, stent fractures, and complete graft disruption) necessitating complex reinterventions with mixed outcomes. The purpose of the present study was to characterize the detection, treatment and outcome of such structural failures in bEVAR BSGs, to assess potential causes and to compare their characteristics with interventions with intact BSGs through the follow-up.

## Materials and methods

Patients with structural failures of BSGs after bEVAR between 2012 and 2020 detected during the follow-up after initially successful BSG deployment were identified in a multi-center bEVAR complication database, consisting of 3 centers. In a retrospective case–control study, the patient files, records of implanted products, outpatient protocols and all pertinent imaging data were retrieved from each respective patient. For inferential analysis, the bEVAR procedures with and without BSG failure from the largest contributing center, regarding bEVAR procedures in general, as well as failure occurrence, were compared. The study was approved by the institutional ethics committee.

### Implantation details

All patients underwent preoperative computed tomography (CT) angiography (CTA) and application of anatomical suitability criteria: Up to 4 essential target vessels, celiac and superior mesenteric artery diameter 6–10 mm, renal artery diameter 4–8 mm, > 25 mm patent aortic lumen at target vessel level, branches located within 50 mm of target vessel ostium, sufficient iliofemoral access vessel diameter, adequate brachial vascular access.

All procedures were performed in general anesthesia with uni- or bilateral femoral access either percutaneously with suture-mediated closure devices (Proglide, Abbott Vascular, Chicago, IL) or surgical exposure of the common femoral artery.

Either off-the shelf (T-branch, Cook medical, Bloomingdale, IL; E-nside, Jotec) or custom-made (CMD, Cook; e-Xtra, Jotec) branched stentgrafts were employed. Proximal thoracic and distal tubular or bifurcated extensions were used as indicated. Brachial access was used in most cases for BSG implantation, alternatively, a steerable sheath (Heli-FX, Medtronic, Minneapolis, IN) was used for complete femoral bEVAR. In cases of insufficient BSG length, an extension BSG was inserted, and in cases of severe angulation or kinking of the BSG, an inner bare nitinol (relining) stent was deployed at the operator´s discretion.

Type, length, and diameter of each BSG were retrieved from the intervention protocols. The maximal angle of the curve in each BSG was measured in the completion angiography images. Post-procedure, all patients received CTA at discharge, 6 months post-procedure, and yearly thereafter. The CTA was performed on multislice scanners with a collimation between 0.6 and 1 mm after i.v. contrast application in the arterial and venous phase. Multiplanar reformatations were available as needed. All CTA images were critically reviewed by an experienced interventional radiologist and screened for signs of BSG failure. Structural BSG failure was considered proven with either contrast extravasation exclusively adjacent to the BSG at CTA and/or if angiographic images with either a clear contrast jet exiting the BSG membrane (absent after repair with a second BSG) or direct wire passage through the BSG were documented angiographically. All CT scans of patients with manifest BSG failure were reassessed by 2 radiologists (S.H., M.F.) and classified according to the following outcome definitions:

### Outcome definitions

BSG *structural failure* was defined as:

- Failure type 1: Fabric disruption with visible endoleak.
- Failure type 2: Fracture of the metallic stents in association with endoleak.
- Failure type 3: Complete structural separation of the stent in two parts.

BSG *failure detection* was classified into one of three modes:

- Detection mode 1: Detection by CT scan with clear signs of BSG fracture.
- Detection mode 2: After one or more CT scans classified as unspecific or type II endoleaks, BSG failure was confirmed at angiography.
- Detection mode 3: After negative CT scan (at the respective branch) as incidental finding during angiography (performed either during repair of a different BSG or main body extension).

### BSG types

The choice of BSGs has evolved over the study period and was influenced by device availability, sheath size, cost, and observations of BSG failures.

The **Advanta V12** (Getinge, Goteborg, Sweden) is a balloon-expandable stainless steel stent encapsulated in a dual extruded polytetrafluorethylene (ePTFE) layer.

The **BeGraft** and **BeGraft Plus Peripheral** (Bentley InnoMed, Hechingen, Germany) are comprised of an inner cobalt chromium stent covered by an ePTFE outer membrane. The membrane thickness was initially 0.1 mm and has been increased to 0.2 mm in 2015. In 01/2018, the BeGraft Peripheral Plus was introduced and incorporated two ePTFE-Stent systems inside each other, thus there are four layers from the outside: ePTFE—cobalt/chrome—ePTFE—cobalt/chrome.

The **E-ventus BX** (Cryolife/Jotec, Hechingen, Germany) consists of a cobalt chromium bare stent with an outer ePTFE membrane, similar to the BeGraft.

The **Viabahn VBX** stentgraft (W.L Gore & associates, Flagstaff, AZ) consists of a stainless steel balloon expandable stent structure, fully surrounded by ePTFE. The inner layer of the membrane is coated with Carmeda bioactive heparin surface with the goal of preventing stent thrombosis. The stent rings are not connected by metal but only by the polymer.

The **Viabahn** stentgraft (W.L Gore & associates, Flagstaff, AZ) is a self-expanding nitinol stent with a Propaten heparin-coated inner ePTFE membrane.

### Statistical analysis

Continuous variables are given as mean ± standard deviation (SD). Categorical variables are given as absolute values and percent (%) for each group. Variables were tested using the Shapiro–Wilk-test for normal distribution. Levene’s test was used to check for equality of variances. Binary variables were analyzed with Fisher´s exact test. A two-sided independent samples t-test with 95% confidence intervals (CI) for normally distributed data, or the Mann–Whitney U-test were used for group comparison in regard of data distribution. Calculations were performed in SPSS 27.0 (IBM, Armonk, NY, USA). A *p* < 0.05 was considered significant.

## Results

**Fig. 1:**
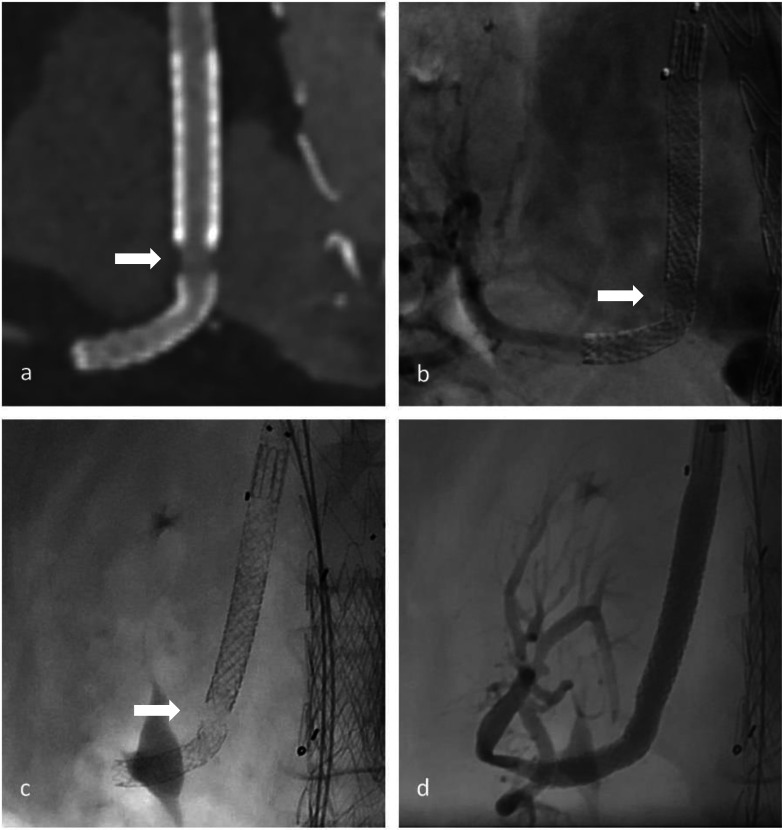
Complete separation of right renal BSG with endoleak directly diagnosed at CTA (**a**), during angiography (**b**, **c**) and after successful religning (**d**)

**Fig. 2:**
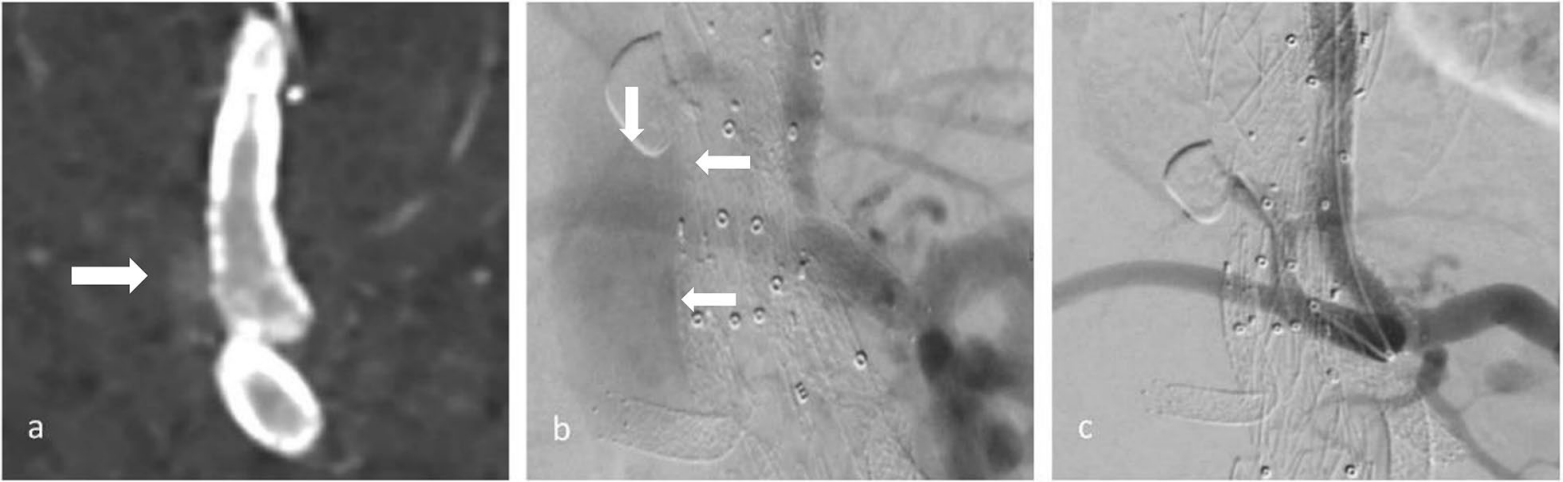
Membrane disruption of BSG in celiac trunk with visible endoleak in CTA (**a**) and angiography before (**b**) and after religning (**c**)

**Fig. 3:**
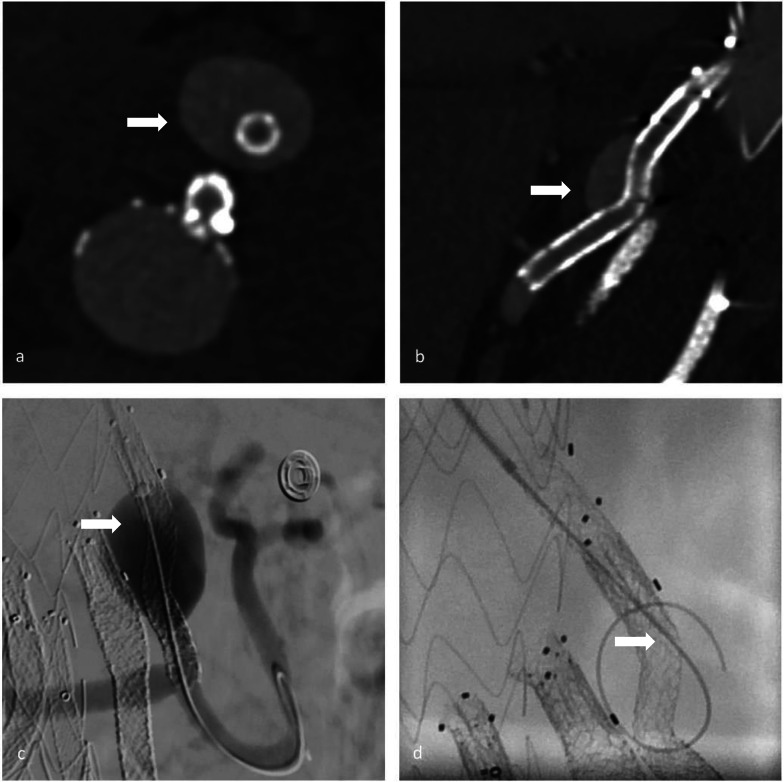
Membrane disruption and wire fracture of BSG (failure mode 2) in celiac trunk with an endoleak surrounding the BSG in CTA (**a**, **b**) and angiography (**c**). Guide wire passage was possible through the fractured BSG into the aneurysm (**d**)

**Fig. 4:**
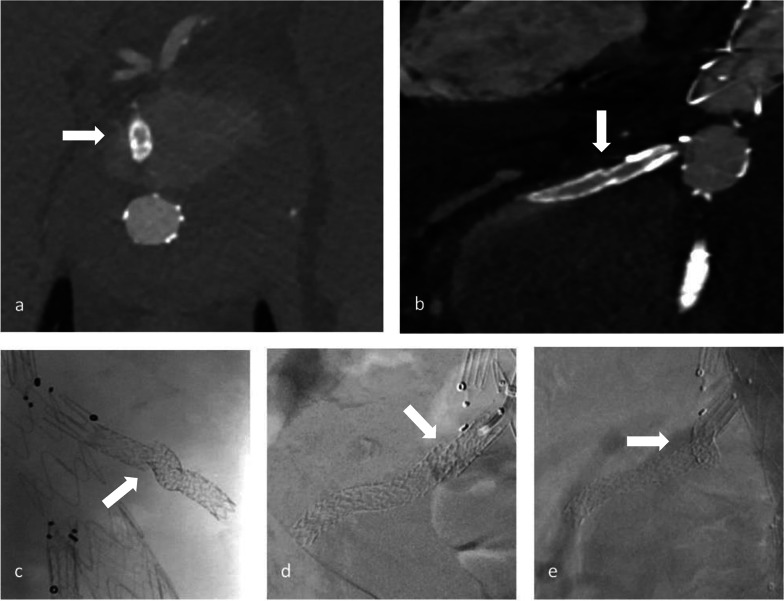
An unspecific endoleak in CTA (**a**) was found to be a complete separation of the SMA BSG in angiography (**c**–**e**). Only after retrospectively calculated CT reformatations (**b**), the BSG fracture is visible. During catheterization, the dislocation of the distal fragment precluded endovascular repair

**Fig. 5:**
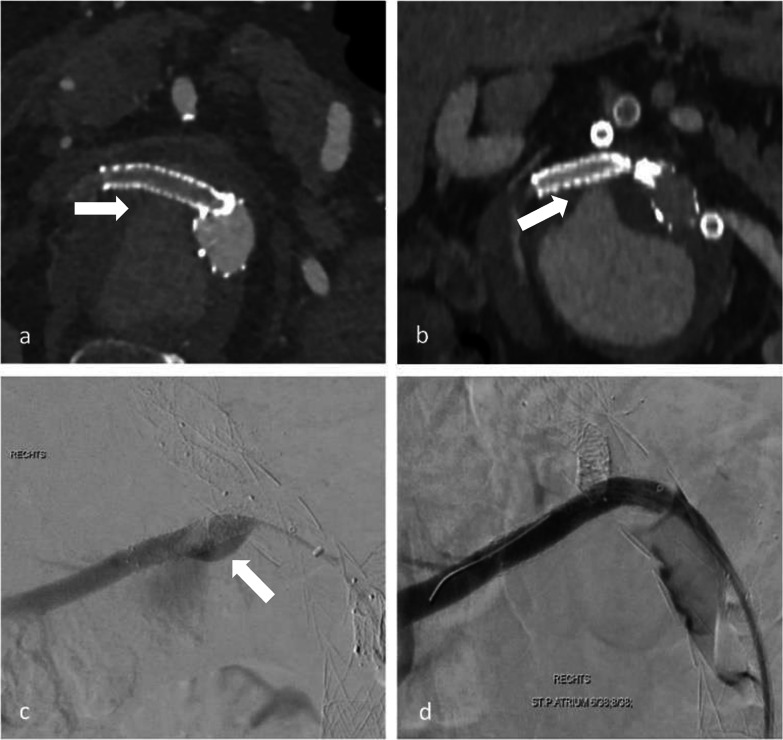
Detection of an unspecified endoleak directly adjacent to the right renal BSG in arterial and venous CTA (**a**, **b**), confirmed to originate from a membrane disruption via angiography (**c**) and after successful religning (**d**)

**Fig. 6:**
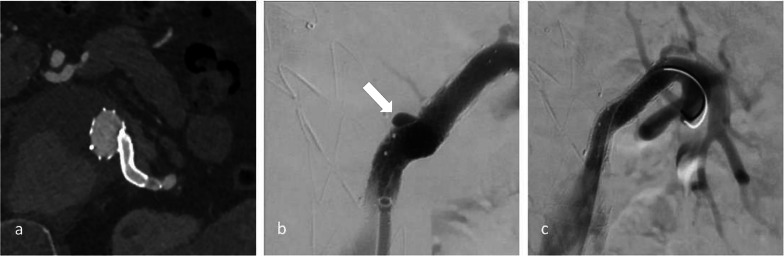
In the same procedure as in Fig. 1, the left renal BSG was unsuspicious in CTA (**a**), but an incidental membrane disruption was detected at angiography (**b**) and successfully treated (**c**)

**Fig. 7:**
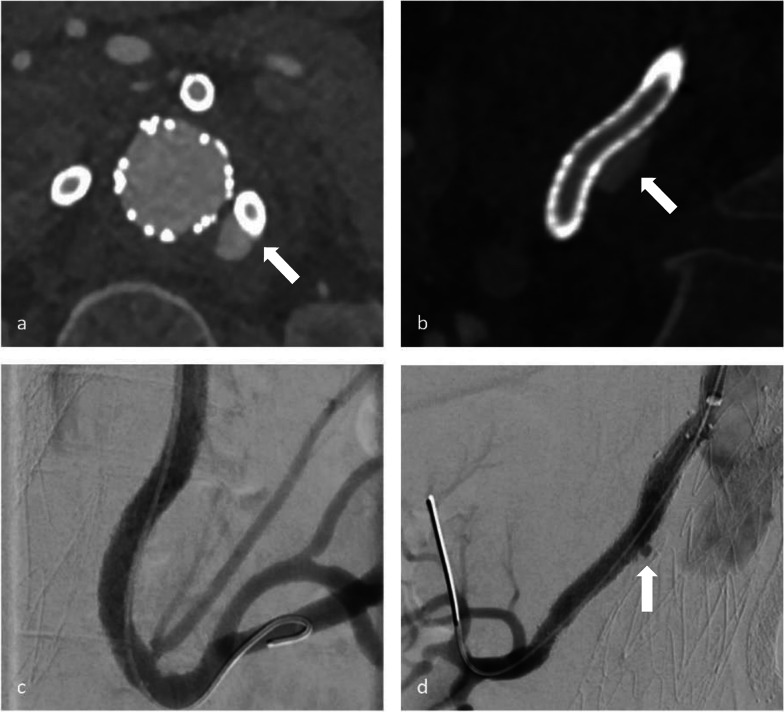
Endoleak adjacent to the left renal BSG in CTA (**a**, **b**). While the endoleak could not be confirmed in angiography (**c**), a previously undetected endoleak of the right renal BSG was found (**d**). Both BSGs were successfully religned. BSG = bridging stentgraft, CT = computed tomography, CTA = computed tomography angiography

Out of 185 branches in 54 patients, we identified a total of 23 BSGs in 12 patients that were affected by structural failure after an initially successful implantation without defects occurring during the implantation (such as e.g., visible perforation during the application of stent in stent or in a religning procedure with a self-expandable stent). According to the grading system regarding the clinical significance of device failures by Chaikof et al., all of the device failures resulted either in the need of intervention (grade 2), or major complication or death (grade 3) [16]. Images of the respective failure modes before and after repair are shown in Figs. 1, 2 and 3. Detection of structural BSG failure occurred after a mean follow-up time of 21.44 ± 15.24 months after the index procedure. Detection mode 1 (clear signs of failure in CT) occurred in 10 BSGs (Figs. 1, 2, 3), detection mode 2 (undefined endoleak at CT confirmed at angiography) in 8 BSGs (Figs. 4, 5), and detection mode 3 (incidental at angiography after negative CT) in 5 BSGs (Figs. 6, 7). The failure types (see definition in Methods) were failure type 1 in 7 BSGs, failure type 2 in 12, and failure type 3 in 4. The affected branches were celiac (*n* = 4), SMA (*n* = 9) and renal (*n* = 10).

All but one BSG failures underwent angiography and an endovascular repair attempt after a mean time of 24.27 ± 39.40 days after detection (one procedure is still pending at the time of submission). In 20/22 BSGs (11 patients), successful endovascular deployment of a second BSG could be performed via a brachial approach with one (*n* = 7) or two (*n* = 2) Advanta V12 and one (*n* = 1) or two (*n* = 2) Begraft (Plus) and two (*n* = 7) or 3 (*n* = 1) Viabahn VBX. In one patient, a complete separation (failure type 3) of a right renal BSG could not be catheterized and the branch was intentionally plugged resulting in loss of the right kidney. In the same patient, another failure type 3 of the mesenteric BSG (again with impossible catheter passage) necessitated open surgery with iliomesenteric bypass. However, procedure-related complications included two mortalities, one from stentgraft-infection with multi-organ failure 13 months after endovascular BSG repair, the other from postoperative pneumonia after iliomesenteric bypass 4 days after BSG repair, as well as one loss of organ (kidney). The other patients had stable outcomes after BSG repair for the remainder of the follow-up of 41.7 ± 26.1 months.

For comparison purposes, the failed and intact BSG groups from the largest contributing center were assessed. Patients with BSG failure were younger and had more reinterventions prior to BSG failure (Reinterventions on other failed BSGs, or extensions of the main body due to endoleak). The main body for bEVAR was similar between groups with predominantly Jotec main bodies. However, failure occurred in 9/26 (35%) E-ventus and BeGraft BSGs, while 4/47 (8.5%) Advanta V12, and 0/19 Viabahn and BeGraft plus failures were detected. There was no difference in the percentage of branches religned with a bare nitinol stent (77 vs. 71%) and side branch extensions with an additional BSG (54 vs. 41%). Failed BSGs were shorter (55 vs 58 mm) and the flexion angle within the branch was larger (50° vs. 28°). Importantly, only four patients had one BSG failure, while 11 BSG failures occurred after one or more previous BSG failures in the same patient.

Tables 1 and 2 show patient and branch characteristics of the respective groups in detail. Table 3 shows detection and treatment details of the multicenter failed BSG group.

**Table 1:** Baseline demographic and clinical characteristics of study population

	BSG failure from database (*n* = 12 patients)	BSG failure cohort single center (*n* = 6 patients)	Control cohort single center (*n* = 25 patients)	*p* (CI of difference)*
Mean age, y	67.25 (± 9.22)	61.73 (± 8.39)	71.84 (± 9.4)	0.022 (1.5; 18.7)
Male sex	10 (83.3%)	5 (83.3%)	15 (60%)	0.383
Comorbidities				
Hypertension	11 (91.7%)	5 (83.3%)	24 (96%)	0.355
Hyperlipidemia	10 (83.3%)	6 (100%)	20 (80%)	0.553
Diabetes mellitus II	3 (25%)	2 (33.3%)	7 (28%)	1.000
Smoking	4 (33.3%)	2 (33.3%)	9 (36%)	1.000
COPD	4 (33.3%)	2 (33.3%)	8 (32%)	1.000
CKD	5 (41.7%)	3 (50%)	7 (28%)	0.358
Atrial fibrillation	0 (0%)	0 (0%)	5 (20%)	0.553
Heart failure	1 (8.3%)	0 (0%)	2 (8%)	1.000
Cancer	1 (8.3%)	1 (16.7%)	5 (20%)	1.000
Arterial disease				
Coronary	5 (41.7%)	3 (50%)	10 (40%)	0.676
Peripheral	1 (8.3%)	0 (0%)	6 (24%)	0.309
Central	0 (0%)	0 (0%)	6 (24%)	0.309
Aortic surgery/intervention priorly	5 (41.7%)	3 (50%)	10 (40%)	1.000
Indication				
TAAA, Crawford:				0.391
I	1 (8.3%)	1 (16.7%)	2 (8%)	
II	4 (33.3%)	2 (33.3%)	6 (24%)	
III	0 (0%)	0 (0%)	0 (0%)	
IV	5 (41.7%)	3 (50%)	15 (60%)	
V	2 (16.7%)	0 (0%)	2 (8%)	
Mean aneurysm Ø, mm	71.00 (± 0.79)	72.33 (± 4.15)	66.12 (± 2.3)	0.247
Urgency				0.654
Acute	0 (0%)	0 (0%)	1 (4%)	
Urgent	2 (16.7%)	2 (33.3%)	4 (16%)	
Elective	10 (83.3%)	4 (66.7%)	20 (80%)	
Index procedure				
Main body				1.000
Jotec	11 (91.7%)	5 (83.3%)	20 (80%)	
Cook	1 (8.3%)	1 (16.7%)	5 (20%)	
Total br, *n*	44	23	85	
Mean br/pt	3.67 (± 0.65)	3.83 (± 0.41)	3.4 (± 0.96)	0.391
Successful br, *n*	44	23	84	
Successful br/pt	3.67 (± 0.65)	3.83 (± 0.41)	3.36 (± 0.95)	0.314
Religning, *n*	17	17	58	
Religning/pt	1.42 (± 1.78)	2.83 (± 1.47)	2.32 (± 1.52)	0.542
Extensions, *n*	18	11	35	
Extensions/pt	1.50 (± 1.17)	1.83 (± 1.17)	1.40 (± 1.63)	0.339
TAM, *n*	35	28	93	
TAM/pt	2.92 (± 2.47)	4.67 (± 2.16)	3.72 (± 2.30)	0.368 (− 3.1; 1.2)
Technical success of BSG				0.330
Primary	10 (83.3%)	4 (66.7%)	22 (88%)	
Assisted	2 (16.7%)	2 (33.3%)	2 (8%)	
Reintervention 30 days	3 (25%)	2 (33.3%)	7 (28%)	1.000
Aortic reintervention (prior to BSG failure)	7 (58.3%)	6 (100%)	9 (36%)	0.007
FU CT/Angiography, months	41.69 (± 26.06)	58.43 (± 26.54)	19.89 (± 34.43)	0.009
FU clinically, months	41.71 (± 26.07)	58.47 (± 26.55)	21.80 (± 34.30)	0.012
BSG related mortality	2 (16.7%)	2 (33.3%)	0 (0%)	0.032

*Br* branches, *BSG* bridging stentgraft, *CI* confidence interval, *CKD* chronic kidney disease, *COPD* chronic obstructive pulmonary disease, *CT* computed tomography, *FU* follow-up, *pt* patient, *TAAA* thoracoabdominal aortic aneurysm, *TAM* total additional material (religning + extensions)

*Between single center cohorts (columns 2 and 3)

**Table 2:** Comparison of branch characteristics and used BSG types in general and for each branch individually

	BSG failure from database (23 branches)	BSG failure cohort single center (13 branches)	Control cohort single center (94 branches)	p*
Main body				1.000
Jotec	21 (91.3%)	11 (84.6%)	77 (81.9%)	
Cook	2 (8.7%)	2 (15.4%)	17 (18.1%)	
Reintervention before	15 (65.22%)	13 (100%)	39 (41.5%)	< 0.001
BSG failure				
BSG				
Br, *n*				0.665
CT	4 (17.4%)	2 (15.4%)	26 (27.7%)	
SMA	9 (39.1%)	3 (23.1%)	27 (28.7%)	
Renal	10 (43.5%)	8 (61.5%)	41 (43.6%)	
Type, *n*				0.004
Advanta V12	4 (17.4%)	4 (30.8%)	43 (45.7%)	
VBX	0 (0%)	0 (0%)	15 (16%)	
E-ventus	16 (69.6%)	6 (46.2%)	14 (14.9%)	
BeGraft	3 (13%)	3 (23.1%)	3 (3.2%)	
BeGraft Plus	0 (0%)	0 (0%)	16 (17%)	
Viabahn	0 (0%)	0 (0%)	3 (3.2%)	
Length, mm	55.78 (± 5.65)	54.62 (± 7.41)	58.02 (± 13.17)	0.010
Ø, mm	7.26 (± 0.96)	7.15 (± 0.90)	7.05 (± 1.14)	0.850
Angle, °		50.46 (± 15.18)	28.36 (± 16.69)	< 0.001
Religning, *n*	10	10	67	
Religning/br	0.44 (± 0.51)	0.77 (± 0.44)	0.71 (± 0.46)	0.672
Extensions, *n*	11	7	39	
Extensions/br	0.48 (± 0.59)	0.54 (± 0.66)	0.41 (± 0.58)	0.502

*CT* celiac trunk, *SMA* superior mesenteric artery

*Between single center cohorts (columns 2 and 3)

**Table 3:** Detection and treatment details of all failed BSG

Detection mode	
1 (Type III EL in CT)	10 (43.5%)
2 (Unspecific EL in CT)	8 (34.8%)
3 (Incidental finding at Angiography)	5 (21.7%)
Time to detection, months	21.44 (± 15.24)
Previous BSG failure	10 (43.5%)
Failure type	
1 (Membrane disruption)	7 (30.4%)
2 (Membrane and wire fracture)	12 (52.2%)
3 (Complete separation)	4 (17.4%)
Treatment, 22 treated, 1 treatment pending	
Religning	20 (90.9%)
Plug	1 (4.5%)
Bypass	1 (4.5%)
Time to treatment, days	24.27 (± 39.40)
Endovascular technical success	20/22 (90.91%)
Complication of intervention	2 (14.3%)

*BSG* bridging stentgraft, *CT* computed tomography, *EL* endoleak

## Discussion

We have observed the structural failure of 23 individual BSGs in 12 patients. All of these structural failures resulted in reinterventions indicated by high-pressure type 3 endoleaks or complete disruption of the stentgraft with disconnection of the arterial supply of the respective visceral organ. These complications not only put the patients at risk of losing the respective organ, but also for aneurysm enlargement or rupture. The reinterventions themselves carried a substantial morbidity and mortality risk.

It is still unclear which factors predispose patients for subsequent BSG structural failure given the low publication incidence and case number. In this study, structural failure tends to occur clustered, as 8/12 patients had failures in more than one BSGs (6 patients in 2 BSGs, 1 in 3 BSGs, and 1 in all 4 BSGs). Contrary to previous suspicions, structural failure exists not only in single layered designs, but in various BSG types used at index procedure [10, 14]: Advanta V12, E-Ventus, and BeGraft. This could indicate that structural failure is, at least in part, contributable to patient-related factors rather than simply attributable to a single manufacturer. The demographics, comorbidities and aneurysm characteristics in the patients with structural failures were not different from other typical EVAR patient cohorts [1, 17]. Potentially certain arterial movements (respiratory or pulsatile) may play a role in premature material degradation, as they have been found to be associated with endoleak formation in conventional EVAR [18]. However, dynamic pre- and postintervention CT studies are not routinely performed. The renal arteries have been reported to be subjected to greater angular movement and torsion than the celiac and mesenteric arteries [15, 19, 20]. However, stent fractures in renal arteries did not occur more frequently in our data set (in 10/23 BSGs) [21]. Potentially, mechanical characteristics of the branch main stentgraft could also contribute to early BSG failure. While BSG failures are slightly overrepresented in the branched Jotec stentgrafts, which were predominantly used in our cohort, larger multicentric data sets are necessary before implications in this direction can be made.

Another factor that may put BSGs at risk for structural failure are prior reinterventions. We observed de-novo BSG failure as early as two weeks after a reintervention. In addition, significantly more prior reinterventions had been performed in BSG failures (either on another BSG or on the main body), compared to intact BSGs (65% vs. 42%, *p* < 0.001). Potentially, the BSGs in some patients are subjected to higher than average stress resulting in the observed clustered failures; however, possible causes for this stress remain as yet unknown.

In addition to the clustered occurrence of BSG failures, we also observed difficulties in their detection and differential diagnosis in routine follow-up CT scans. Only 10 of 23 BSG failures were clearly identified as such at CT, the remainder typically showed signs of endoleak not attributable to a specific source during multiple subsequent CT scans, leading to increased number of investigations and a delay in diagnosis. In 5 of 23 BSGs, CT did not show any endoleak and the failure was detected incidentally during angiography. Given the fact that in the last stage of BSG failure, the complete separation of the components, endovascular repair may prove impossible and subjects the patient to considerable morbidity and mortality, BSG failure detection at an earlier stage seems desirable. It seems advisable to implement shorter surveillance intervals in patients after one BSG failure. Further, bEVAR patients could profit from CT follow-up scans with higher spatial resolution around the aorta at the cost of a reduced field of view as used in cardiac CT protocols [22]. Finally, a more liberal indication to angiography when unspecific endoleaks are reported at CT scans may aid in detecting BSG failures at an earlier stage. On the side of the interpreting radiologist, a clear understanding of the function and weaknesses of BSGs and a tailored CT angiography protocol, potentially with higher resolution and/or time-resolved post contrast series, may aid in earlier detection and less invasive repair [23, 24]. Since 19 of the 23 BSG failures occurred in single-layered stentgrafts, it seems unwise to continue their use in bEVAR, as several double-layer stentgrafts are now readily available, although with larger sheath sizes and higher cost.

Currently there are no stentgraft manufacturers who include the use of their products as BSGs for bEVAR in their instructions for use. Consequently, all types of BSGs for bEVAR are used off-label [14, 15]. Since structural failures were observed with single- and double-layered BSG types, a dedicated BSG would probably need more structural strength and material durability than the currently available products [21, 25]. Further development of stentgrafts is ongoing and yields promising results toward more resilience [26, 27].

In the foreseeable future, bEVAR will be expanded in its indications and in its frequency to an ever-larger patient cohort as a powerful alternative to thoracoabdominal open aortic replacement [28]. Currently there is no candidate for a dedicated BSG announced so we will be faced with an ever-increasing number of off-label use in the necessary BSGs. Alternatively, an existing product with marginal improvements might prematurely be labelled as suitable for this task, but the results of this and similar studies advise caution. Compared with current rates in literature, structural failures are probably underreported [16, 29] and the more widespread use of bEVAR will undoubtedly bring up more BSG failures. While some users have learned to put the blame on the material last, this may not necessarily hold true in this application. Especially BSGs require clinical validation and monitoring as a relatively newly developed technology rather than just relying on bench tests [16].

Stricter quality controls may be in order given the fact that the detected structural failures in BSGs occurred at a mean time of 21 months after insertion. Also, the practice of relining BSGs with self-expanding nitinol stents must be reassessed, as the spikes of the inner stent potentially contribute to early material fatigue. Our data did however not show a trend to more failures in religned BSGs. On the other hand, a greater curve of the BSG was associated with higher failure rates. Thus, an even more watchful eye in postoperative CT surveillance should be turned toward BSGs with significant angulation.

Since the products are used off-label and a relevant chance of failure of the product in this position exists, there are likely consequences regarding legal responsibility and patient-informed consent. Uncertainties in this respect may contribute to a negative publication bias: To report complications of an off-label use with unclear accountabilities may deter some researchers. As bEVAR will continue to be a valuable and necessary treatment option, clear guidelines concerning the necessary off-label use of BSGs are needed. So far manufacturers leave physicians in the cold regarding this aspect.

Limitations of the study include the relatively low event count, even in a multicenter approach. The increased frequency of bEVAR in our center over the last 2 years contributes to the shorter mean follow-up in the control group. Moreover, changes in the used BSG types may distort apparent failure rates across the different manufacturers. Larger registries will be needed to draw inferential conclusions as to which factors contribute to BSG failure and to investigate the clustered occurrence. Hopefully, this publication will create awareness and encourage more uniform reporting standards, thereby facilitating the creation of said registries.

## Conclusion

Structural failures of BSGs after bEVAR with potentially serious consequences occur throughout the available product range. Moreover, they arise in a product that has been intentionally used off-label because there are no dedicated products for this purpose available. Our data show that previous structural failure in one BSG, aortic reinterventions and BSG angulation and the use of single-layer BSGs are risk factors for BSG failure in the same patient. As of yet there are no clear additional indicators which procedures or patients are more prone to structural failure than others. It seems advisable to keep an open eye on early stages of structural failure during the follow-up CT scans after bEVAR and be aware about patient-informed consent regarding BSGs.

## Data Availability

The datasets used and analyzed during the current study are available from the corresponding author on reasonable request.

## Abbreviations

bEVAR: Branched endovascular aortic repair
BSG: Bridging stentgraft
CI: Confidence interval
CT: Computed tomography
CTA: Computed tomography angiography
ePTFE: Extruded polytetrafluorethylene
EVAR: Endovascular aortic repair
fEVAR: Fenestrated endovascular aortic repair
SD: Standard deviation
